# Assessment of prokaryotic communities in Southwestern Atlantic deep-sea sediments reveals prevalent methanol-oxidising Methylomirabilales

**DOI:** 10.1038/s41598-023-39415-9

**Published:** 2023-08-07

**Authors:** Francielli V. Peres, Fabiana S. Paula, Amanda G. Bendia, Júlia B. Gontijo, Michel M. de Mahiques, Vivian H. Pellizari

**Affiliations:** 1 Department of Biological Oceanography, Oceanographic Institute, University of São Paulo, Praça do Oceanográfico, 191, São Paulo, CEP: 05508-120 Brazil; 2 Cell and Molecular Biology Laboratory, Centre for Nuclear Energy in Agriculture, University of São Paulo, Piracicaba, Brazil; 3 Department of Physical, Chemical and Geological Oceanography, Oceanographic Institute, University of São Paulo, São Paulo, Brazil

**Keywords:** Environmental microbiology, Microbial communities, Marine biology

## Abstract

Continental slopes can play a significant contribution to marine productivity and carbon cycling. These regions can harbour distinct geological features, such as salt diapirs and pockmarks, in which their depressions may serve as natural sediment traps where different compounds can accumulate. We investigated the prokaryotic communities in surface (0–2 cm) and subsurface (18–20 or 22–24 cm) sediments from a salt diapir and pockmark field in Santos Basin, Southwest Atlantic Ocean. Metabarcoding of 16 samples revealed that surface sediments were dominated by the archaeal class Nitrososphaeria, while the bacterial class Dehalococcoidia was the most prevalent in subsurface samples. Sediment strata were found to be a significant factor explaining 27% of the variability in community composition. However, no significant difference was observed among geomorphological features. We also performed a metagenomic analysis of three surface samples and analysed the highest quality metagenome-assembled genome retrieved, which belonged to the family CSP1–5, phylum Methylomirabilota. This non-methanotrophic methylotroph contains genes encoding for methanol oxidation and Calvin Cycle pathways, along with diverse functions that may contribute to its adaptation to deep-sea habitats and to oscillating environmental conditions. By integrating metabarcoding and metagenomic approaches, we reported that CSP1–5 is prevalent in the sediment samples from Santos Basin slope, indicating the potential importance of methanol metabolism in this region. Finally, using a phylogenetic approach integrating 16S rRNA sequences assigned to Methylomirabilota in this study with those from a public database, we argued that CSP1–5 public sequences might be misclassified as Methylomirabilaceae (the methanotrophic clade) and, therefore, the role of these organisms and the methanol cycling could also be neglected in other environments.

## Introduction

The continental margins are complex and unique environments that interconnect terrestrial and marine processes, playing a significant role in the biogeochemical cycles of carbon and nitrogen^1^. Although continental shelves and slopes represent only 20% of the ocean area, these regions can account for up to 50% of marine productivity^2,3^. The mineralisation of organic matter in these areas occurs at a much faster rate when compared to what occurs in open sea sediments, allowing the regenerated nutrients to quickly return to their natural cycles^4,5^. Continental slopes often comprise a wide range of physical and geological features that can affect the nutrient distribution and, consequently, biodiversity^6^. This variability is particularly important for benthic microbial communities, which are known to respond to changes in nutrient availability and carbon substrate type^7^. As microbial processes are essential for ocean biogeochemistry and organic matter mineralization^5^, it is crucial to understand their roles in local and regional carbon cycles.

Santos Basin (SB) is a marginal basin located on the Southwestern Atlantic margin. In this region, the continental upper to middle slope presents high declivity and is marked by numerous kilometre-scale seafloor features, including pockmarks and exhumed salt diapirs^8,9^. Pockmarks are crater-like depressions formed by the sudden release of fluid (predominantly methane) in the form of a gas or liquid to the surface^10,11^. In Santos Basin, fluid expulsion is facilitated by moving large volumes of salt, weakening the upper sedimentary layers^9^. Due to gravitational forces, these salt masses show vertical movement (diapir), which may or may not be exposed on the marine sedimentary surface^8,12^. The pockmark field on the SB continental slope has been reported to contain more than nine hundred depressions related to this geological feature and salt diapirs. Although the majority of pockmarks in this area are thought to be not actively seeping gas, acoustic data, and metal proxies have suggested the presence of recent to sub-recent seepage activities in some of the pockmarks^9,13–16^. In addition, the unique characteristics of the seafloor in this area may provide a myriad of habitats for diverse but yet poorly explored microbial communities^17^.

Continental slopes often present high sediment accumulation and organic matter deposition^9,18^. In addition, the depressions as pockmarks may serve as natural sediment traps^16^, where the sedimentation of different compounds can occur, including methanol, a highly abundant reduced carbon in the marine environment^19,20^. This C1 hydrocarbon has a fast turnover in this environment, on the order of a few days, indicating its significance in biological cycles, mainly as a source of carbon and energy for methylotrophic microorganisms^21,22^.

Versatile metabolic capabilities provide the seafloor microbial communities with different strategies to couple with oligotrophic and variable conditions. The importance of C1 metabolism in deep-sea sediments has been recently suggested by Torres-Beltrán et al. ^23^, including methanol oxidation to formaldehyde. The authors also detected functions related to formaldehyde oxidation on the continental slope of the southern Gulf of Mexico. Yet, the identity, metabolic capabilities, and distribution of these organisms demand further exploration. While some methylotrophic groups have been vastly studied, others have only recently emerged from metagenome-assembled genomes (MAGs). Hug et al. ^24^ described a genome of a non-methanotrophic methylotroph from the phylum Methylomirabolota (former NC10), a cluster mostly known for its nitrate-dependent methanotroph members, which has raised questions regarding the phylogenetic boundaries of the different methylotrophic groups in this phylum.

In this study, we used metabarcoding and metagenomics to investigate the prokaryotic diversity in surface and subsurface sediments collected at the pockmark and salt diapir field on the SB continental slope. In addition, through genome reconstruction from metagenomic data, we described the MAG of a methylotroph from the order Methylomirabilales, which was widespread across SB sediments. We further explored its adaptations to the deep-sea environment and then argued its potential metabolic capabilities and roles in the carbon cycle along the SB continental slope.

## Material and methods

### Study area and sediment sampling

The Santos Basin (SB) is located on the southern continental margin of Brazil between latitudes 27° S and 26° N, covering an area of 3.5 × 10^5^ km^2^. SB is limited to the north by Alto de Cabo Frio and to the south by Cabo Santa Marta Grande in Alto de Florianópolis^25^. Under the hydrographic point of view, the area of study is localised in the transition between two water masses, the South Atlantic Central Water (SACW) and the Antarctic Intermediate Water (AAIW) ^33^. The SACW (T ≤ 18.5 °C, S ≥ 35.3) occupies the pycnocline level, while the AAIW presents temperatures between 3 and 6 ^o^C and salinities between 34.2 and 34.5^26–28^.

**Figure 1:**
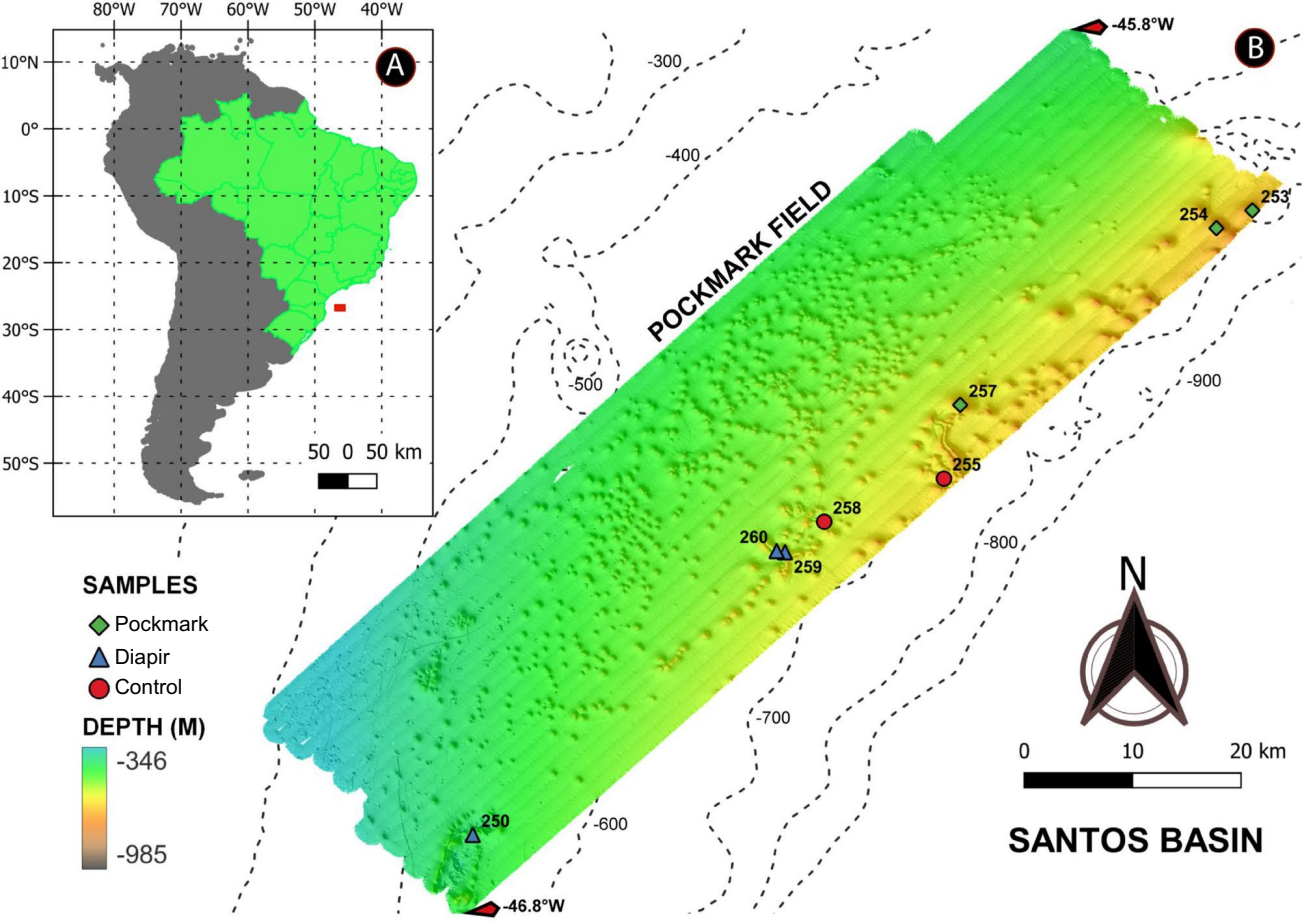
Map of the study region located in the Santos Basin, highlighting the sampling sites where structures related to pockmarks and salt diapers were found on the Brazilian continental margin.

We collected sediment samples using a stainless-steel box corer BX-650 (Ocean Instruments, San Diego, California, USA) (50 cm × 50 cm) with a maximum penetration of 60 cm. Cylindric corers were used to collect sediments within the box corer while maintaining the sediment stratification. The sediment cores were sliced into 2 cm layers with sterile spatulas and placed in whirl pak bags, then stored at −20 °C until processing. For this study, we selected eight stations with bathymetry ranging from 400 to 800 m approximately, comprising three stations located in salt diapirs, three stations in pockmarks, and two stations in the adjacent marine seafloor considered as control sediments, without pockmarks and salt diapirs influence (Fig. 1). We used the superficial (0–2 cm) and the deepest sediment layer (16–18 or 22–24 cm), from now on called surface and subsurface strata, respectively (Table S1).

### Taxonomic profiling of the prokaryotic community

DNA was extracted from 0.25 g of sediment using the Power Soil DNA Isolation Kit (Qiagen, Germany), following the manufacturer's specifications. DNA integrity was verified by electrophoresis in 1% (v/v) agarose gel, and concentration was assessed using the Qubit dsDNA HS assay kit (Thermo Fisher Scientific, Waltham, MA, USA). The V4–V5 hypervariable region of the 16S rRNA sequence was amplified with the universal primers 515F and 926R^29,30^. The initial PCR reaction consisted of a denaturation step at 95 °C for 3 min, followed by 35 cycles with 95 °C for 30 s, annealing at 57 °C for 30 s, extension at 72 °C for 30 s, and final extension at 72 °C for 5 min. Library preparation and sequencing were performed by Mr DNA/Molecular Research (Shallowater, TX, USA), using Illumina Miseq platform (2 × 250 bp system). 16S rRNA sequencing data are available in the National Center for Biotechnology Information Sequence Read Archives under BioProject ID PRJNA818533.

After sequencing, paired-end reads were initially imported and demultiplexed into the QIIME2 software version 2019.10^31^. Graphic inspection of quality profiles was performed, low-quality reads (below Phred score 30) were trimmed, and the chimeras were removed with the aid of the Dada2 software^32^. After quality control, the amplicon sequence variants (ASVs) were determined using the Dada2 software into the QIIME2 package. Taxonomy was assigned through feature-classifier classify-sklearn and SILVA database v. 138. ASV richness, Chao1, and Shannon diversity indexes were calculated using *phyloseq*^33^ and *vegan* packages^34^, and *ggplot2*^33^ was used for graphing in R v. 4.1.0 (R Development Team, 2018). The normality of variances was assessed by Shapiro–Wilk test. Similarities among samples and site groups were examined using ordinated weighted Unifrac normalised distance and visualised by non-metric multidimensional scaling (NMDS). Alpha diversity, statistical analysis, and data visualisation were carried out in R using the *Phyloseq* package^33^. Differences in the microbial communities among sites and depths were assessed by permutational multivariate analysis of variance (PERMANOVA)^35^.

### Metagenomic analysis for metagenome-assembled genome (MAG) recovery

We performed shotgun metagenomic analysis of three surface sediment samples from the following stations: 259 (diapir area), 260 (diapir area), and 255 (control area). The metagenomic libraries were prepared using Nextera XT DNA Library Prep Kit (Illumina, San Diego, CA, USA), and sequencing was performed in Illumina Hiseq platform (2 × 150 pb system) at the Woods Hole Institute's Marine Biology Laboratory, as part of the “Deep Carbon Observatory's Census of project Deep Life”. Raw reads were filtered through the SICKLE software with phred > 30 and then used for genome reconstruction through the anvi’o pipeline v. 7.1^34^. Co-assembly was performed using the Megahit software^36^, and the contigs with size > 4000 bp were selected for binning through the CONCOCT software^37^. Bins were manually refined using *anvi-refine*^34^ and then quality checked with CheckM v. 1.0.7^38^. MAGs were taxonomically classified based on genome phylogeny according to the *classify* workflow (classify_wf) from the Genome Taxonomy Database Toolkit (GTDB- Tk v. 1.3.0) and the Genome Taxonomy Database (GTDB; release 202)^39^. Metagenome raw sequences are available in the GenBank repository under BioProject ID PRJNA818670.

For further analyses, we selected the MAG with the highest quality scores, the SB_MAG_00001 (94.2% completeness and 2.1% contamination), classified within the Methylomirabilota phylum (formerly NC10 phylum). Prediction and annotation of ORFs were performed using prokka v.14.5^40^. Ghost-KOALA (genus_prokaryotes) and SEED Subsystem through RASTtk^40^ were used for functional annotations of the predicted protein sequences. MetabolismHMM tool v. 1.9 (https://github.com/elizabethmcd/metabolisHMM) and DRAM (Distilled and Refined Annotation of Metabolism) tool v. 1.2.4 (https://github.com/shafferm/DRAM) were used to annotate genes related to sulphur, nitrogen, and carbon metabolisms. The coverage and relative abundance of the MAG in relation to its co-assembly sequence library was estimated using Bowtie2 2.3.2^41^. The MAG was deposited in Figshare under https://doi.org/10.6084/m9.figshare.20080031.

### Construction of the phylogenetic tree

A phylogenetic tree was built to verify the phylogenetic relationships among members of the Methylomirabilota phylum recovered from our sediment samples through metabarcoding and metagenomics. First, we selected all sequences assigned to this phylum found in our 16S rRNA sequencing data (23 ASVs), and then extracted the 16S rRNA sequence from the MAG SB_MAG_00001 using the barrnap software (version 0.9, https://github.com/tseemann/barrnap). In addition, 16S rRNA sequences were extracted from three reference genomes from the order Methylomirabilaliles, available in the NCBI (National Center for Biotechnology Information): Candidatus *Methylomirabilis oxyfera* (Ca. *M. oxyfera*) (NCBI:txid671143), Candidatus *Methylomirabilis limnetica* (Ca. *M. limnetica*) (NCBI: txid671143), and NC10 bacterium CSP1-5 (NCBI:txid1640516). Finally, we used all 16S rRNA sequences available in the SILVA database that showed at least 95% identity with our Methylomirabilota 16S rRNA sequences and the 16S rRNA sequence extracted from our MAG SB_MAG_00001 (34 sequences). The reference sequences from SILVA database were retrieved from marine and terrestrial ecosystems.

All sequences were aligned using the Mega X software^42^ through the clustalW algorithm that uses progressive alignment methods^43^. This algorithm calculates an approximate distance matrix between pairs of sequences based on alignment scores. The phylogenetic tree was constructed by the maximum likelihood method with Bootstrap replications equal to 999.

## Results and discussion

### Microbial taxonomic diversity in sediments from the Brazilian continental slope

The 16S rRNA gene sequencing of the 16 samples yielded a total of 573,850 quality-filtered reads, divided into 11,034 amplicon sequence variants (ASVs) (0.03 cut-off). Of those, 9244 ASVs were assigned to Bacteria and 1788 to Archaea. Richness and diversity assessed using Chao1 and Shannon indices (Table S2), did not change significantly across the types of seabed sampled (control, diapir, pockmark) and between the two sediment strata (surface and subsurface strata) (Table S3).

**Figure 2:**
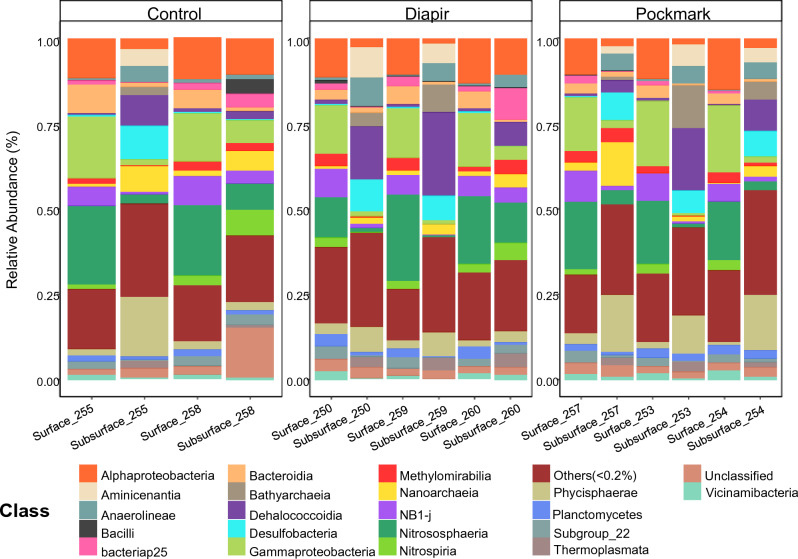
Bar graphs showing the relative abundance of bacterial and archaeal classes in the Control, Diapir and Pockmark sediment samples collected in the Santos Basin. Surface = 0–2 cm and subsurface = 16–18 cm or 22–24 cm.

Surface sediments were dominated by the archaeal class Nitrososphaeria (Crenarchaeota) across all seabed types, accounting for up to 25% of the taxonomic assignments (Fig. 2). Nitrososphaeria is one of the most widely distributed classes of Archaea, found in a diversity of environments^44,45^, including marine water and sediment^46,47^. It comprises chemolithoautotrophic ammonia-oxidising taxa, which play an important role in the marine nitrogen and carbon cycles^48^. Alpha and Gammaproteobacteria comprised between 11 and 18% of the ASVs assignments. These classes have also been reported as part of the dominant groups in benthic marine environments, including pockmark sediments^45,49–52^.

The class NB1-J, previously described in sediment samples from active pockmarks^52^, was detected in surface sediments from the SB. The class Methylomirabilia, from the phylum Methylomirabilota (former NC10), was found in all surface samples, with an average abundance of approximately 2.6%. Members of this class have been identified in the most diverse environments, from freshwater to saline, and include methanotrophic and non-methanotrophic methylotrophic taxa^24,53,54^.

In the subsurface samples, the Dehalococcoidia represented up to 24% of the communities. Some members of this class can perform organohalide respiration, anaerobic respiration that uses halogenated organic compounds as terminal electron acceptors for hydrogen oxidation^55^. Microbial dehalogenation plays a significant role in the functioning of the halogen cycle, and many of these organisms are found in the marine environment, mainly in subsurface sediments^55,56^. The class Phycisphaerae (phylum Planctomycetota) was also prevalent across all subsurface samples from SB, reaching up to 17% of the communities. These organisms are aerobic or facultatively anaerobic and colonise a wide variety of ecosystems, from aquatic to terrestrial and even extreme environments such as desert, saline, and thermal soils^57–60^.

Desulfobacteria, a class known to contain sulphate-reduction bacteria, a key function in the sulphur cycling and anaerobic respiration^61^, presented relative abundance between 0.2 and 9.8% of the subsurface communities. The Methylomirabilia class, found in all analysed samples, represented approximately 1.5% of the communities in the subsurface samples. Nitrososphaeria comprised a much smaller proportion in the most profound strata than in the surface samples. By contrast, another Crenarchaeota class, the Bathyarchaeia, was found in higher proportions in the subsurface sediments. These organisms are widely distributed and abundant in marine sediments; however, the environmental factors that control their distribution are currently unclear^62^. They are metabolically diverse and indicated by Lazar et al. ^63^ as degraders of aromatic compounds and recalcitrant organic matter. The acetogenic ability to lithotrophically synthesise acetate from inorganic carbon has also been suggested through MAGs studies, as well as the potential ability to metabolise methane; however, these metabolisms were not yet confirmed by physiological studies since they were not yet cultivated in laboratory conditions^53,64^.

**Figure 3:**
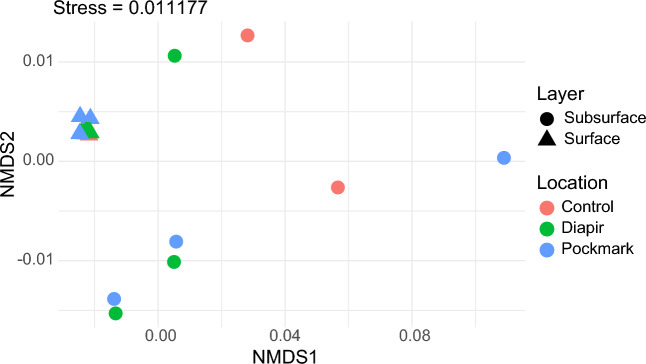
Non-metric multidimensional scale (NMDS) of the surface (0–2 cm) and subsurface (16–18 cm or 22–24 cm) sediment communities. Stress = 0.011177. Geometric shapes indicate sediment strata and colours indicate collection sites.

According to PERMANOVA analysis, seabed type was not a significant factor affecting the composition of the communities (Table S4). By contrast, 27% of the community variability (p < 0.001) was explained by the vertical sediment strata—surface (0–2 cm) versus subsurface (16–18 cm or 22–24 cm). These findings agree with previous studies showing remarkable differences in prokaryotic communities along the sediment strata, which is attributed to the sharp changes in physical and chemical conditions with depth^17,49,65,66^. Another interesting pattern was revealed by NMDS ordination, where all surface sediment communities were concentrated in a tight cluster, indicating high similarity in their composition, while lower similarity was observed among subsurface samples (Fig. 3). As surface sediments are under direct contact with the pelagic environment, their biotic and abiotic characteristics suffer stronger influences from the recent deposition processes and exchanges with the water column. This aspect also contributes to the higher availability of organic carbon^67,68^. In addition, high-energy electron acceptors such as oxygen and nitrate are often available in the surface sediments^69^. By contrast, deeper sediment layers may reflect events during the deposition, such as different sedimentation rates^70^, which may vary along the continental slope^71,72^.

The core microbiome analysis further supported these contrasting patterns for the community similarity when comparing surface and subsurface strata. In this analysis, we asked whether there would be ASVs common to all samples within each stratum, as well as to all samples regardless of the stratum (central core microbiome). While 80 ASVs were shared by all surface sediment samples (surface core microbiome), comprising archaeal and bacterial taxa from ten different phyla, the core microbiome from the deep strata was composed of only two ASVs, which were also the central core microbiome, i.e., present in all samples (Table S5). These two ASVs were assigned to the Methylomirabilaceae and Hyphomicrobiaceae families, according to SILVA database v.138. In two recent studies carried out in Santos Basin deep-sea sediments, the phylum Methylomirabolota was reported among the dominant groups across different bathymetries (from 450 to 1250 m)^17^, and in methane-enriched areas^73^. The Hyphomicrobiaceae family (Alphaproteobacteria) has been identified in several marine and non-marine habitats^74,75^ and are morphologically and physiologically diverse, with most of their members known to perform aerobic chemoheterotrophic metabolism. However, some representatives, such as members of the genus *Hyphomicrobium,* can grow anaerobically by denitrification or fermentation^75^. Interestingly, as the Methylomirabilales, the family Hyphomicrobiaceae also comprises methanol assimilating representatives^24,76,77^, suggesting that C1 metabolising organisms could be widespread in the studied area. However, there are still many gaps in knowledge about the dynamics of methylotrophic microorganisms and their metabolic capabilities in deep-sea sediments.

### SB_MAG_00001: a Methylomirabilales methylotroph retrieved from SB surface sediment samples

The metagenomic library constructed from three surface sediment samples yielded 77,922,888 raw reads. After quality filtering, the number of reads per sample was 23,406,011 (Surface_255), 19,431,982 (Surface_259) and 22,090,471 (Surface_260). The co-assembly resulted in 108,018 contigs (> 1000 bp, N50 = 1492 bp), which were further binned into 34 MAGS: two high-, six medium-, and 26 low-quality drafts, according to genome quality standards suggested by Bowers et al. ^78^ (Table S6). SB_MAG_00001 exhibited the highest quality parameters (completeness of 94.2% and contamination of 2.1%), and represented 0.4% of mapped reads (30× coverage). According to the phylogenomic analysis of the GTDB-Tk, this MAG was assigned to the order Methylomirabiles, family CSP1-5 (ANI of 97.77% with CSP1-5 sp001443495 as the closest representative genome).

Considering the data presented, it is noteworthy that contrasting taxonomic assignments were observed for members of the Methylomirabilales order retrieved by the different sequencing approaches in our study. While using SILVA v.138 database to classify 16S rRNA sequences, all ASVs from the Methylomirabilales order were assigned to the family Methylomirabilaceae (including one of the two ASVs from the central core microbiome). By contrast, the genome of SB_MAG_00001 was classified within the family CSP1-5, according to GTDB-Tk. Although Methylomirabilales is still a poorly known taxon, this difference in taxonomy may have important implications for our conclusions regarding microbial function and carbon cycling, as these families have distinct key metabolic capabilities.

To date, there is no isolated culture from the order Methylomirabilales. The most studied organisms belong to the family Methylomirabilaceae (Ca. *Methylomirabilis oxyfera*, Ca. *M. limnetica*, and Ca. *M. lanthanidiphila*), which are known to carry out anaerobic oxidation of methane (AOM) coupled to nitrite reduction^54,79,80^. Unlike other AOM processes, they employ enzymatic machinery similar to aerobic methanotrophy, including its central enzyme, the methane monooxygenase. The oxygen required for this process is produced intracellularly by nitric oxide (NO) disproportionation^80^. However, this methane oxidising enzyme complex is absent in CSP1-5, a genome retrieved from aquifer sediment samples^24^, and characterised as a non-methanotrophic methylotroph. The CSP1-5 genome shares 89% of the 16S rRNA sequence identity with Ca. *M. oxyfera.* The phylogenetic differences described between CSP1-5 and the Ca. *M. oxyfera* establish the boundaries of denitrification coupled with methane oxidation within the Methylomirabilota phylum^24^.

**Figure 4:**
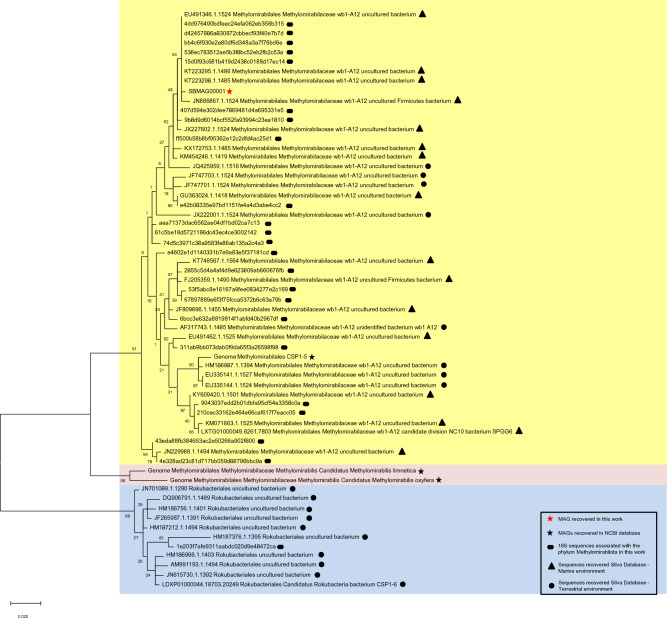
Phylogenetic tree comparing the 16S sequences affiliated to the order Methylomorabilales obtained in our samples, together with the 16S sequence recovered from SB MAG 00001. The analysis also includes 16S sequences recovered from the reference genomes: Ca. *Methylomirabilis oxyfera*, Ca. *Methylomirabilis limnetica* and NC10 bacterium CSP1–5, obtained through data deposited at NCBI (National Center for Biotechnology Information). In addition, 16S rRNA sequences retrieved from the Silva 138 database, with at least 95% similarity with the sequences from this study, were also used.

By inspecting the SILVA database (v.138), it is possible to verify that Methylomirabilaceae is the only family registered within the order Methylomirabilales in their library. Therefore, we asked whether the CSP1-5-like microorganisms in our samples could have been misclassified by the SILVA database. To answer this question, a phylogenetic tree was built using the SB 16S rRNA amplicon sequences classified into the Methylomirabilota phylum and the SSU fragments extracted from SB_MAG_00001 and from three reference genomes: Ca. *M. oxyfera, Ca. M. limnetica* and CSP1-5. In addition, 16S rRNA sequences downloaded from SILVA were also used (see methods for details). According to Ettwig et al. (2009)^81^, the phylum Methylomirabolota can be divided into four groups: *A*, *B*, C, and *D*. Groups *A* and *B* are considered the dominant branches and harbour the anaerobic methane-oxidising microorganisms. Members of the CSP1-5 family are housed in the group *D*. All other members of this phylum are allocated to clade *C*^81^. In the phylogenetic tree presented in Fig. 4, the 16S rRNA sequences clustered in three major clades: One clade was formed only by sequences extracted from the reference genomes of the methanotrophic taxa, Ca. *M. oxyfera* and Ca. *M. limnetica* (both group A)^54,81^. The largest cluster housed 22 ASVs obtained in this study by amplicon sequencing and the sequences extracted from the genomes of SB_MAG_00001 and CSP1-5. Therefore, the clustering pattern indicates that the SB amplicon sequences are more closely related to CSP1-5 than the methanotrophic genomes. This finding suggests that those sequences may have been misclassified by the SILVA database and could, in fact, belong to the same family as SB_MAG_00001, CSP1-5. In addition, this large cluster also contained 34 sequences downloaded from the SILVA database (retrieved from marine and terrestrial ecosystems) and classified within the family Methylomirabilaceae. According to this analysis, previous studies may have inaccurately reported the presence of methane-oxidising microorganisms and neglected the importance of methanol metabolism in those environments (Table S7). The third cluster comprised ten sequences from the SILVA database and only one ASV retrieved from this study by amplicon sequencing. All members of this cluster belong to the order Rokubacteriales. Interestingly, except for the ASV obtained in this study, all sequences in the third clade were recovered from terrestrial environments.

### Potential metabolisms and lifestyles of the SB_MAG_00001

SB_MAG_00001 contains complete or nearly complete gene sets for the following central carbon and energy metabolism pathways: glycolysis, gluconeogenesis, pyruvate oxidation, TCA cycle, pentose phosphate pathway, and Calvin Cycle. The MAG contains genes for the lanthanide-dependent Xox-type methanol dehydrogenase (MDH), which can convert methanol to formaldehyde or directly to formate^82–84^. It has also been proposed that XoxF-MDH can oxidise formaldehyde to formate^83^. Several studies have suggested the widespread nature of Xox-MDH within methylotrophs, with its functional relevance in the environment likely comparable with the vastly studied calcium-dependent MDH (Mxa-MDH).

**Figure 5:**
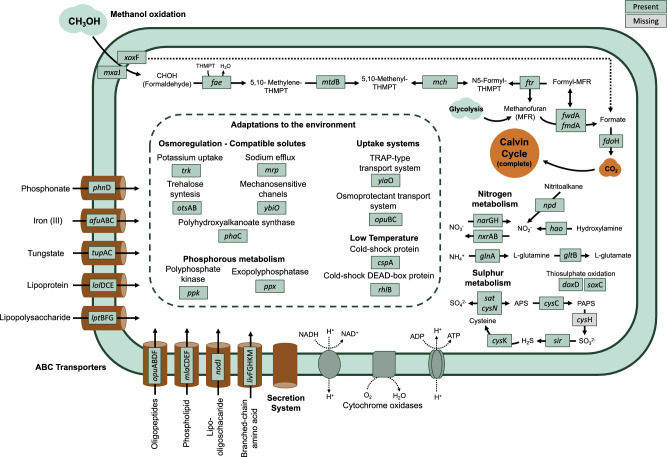
Model prediction of SB_MAG_00001 metabolisms. The model includes potential capabilities related to methanol, nitrogen (N) and sulphur (S) metabolisms, as well the ABC transporters and the adaptations to the environment. The model was constructed with the genes annotated by DRAM, predicted proteins annotated by GhostKoala and the hidden Markov model (HMM) searches (Supplementary Table 8).

The metabolic reconstruction in Fig. 5 presents the MAG's complete pathway to oxide formaldehyde to formate through the 5,6,7,8-tetrahydromethanopterin-dependent route, in addition to formate dehydrogenase, which can oxidise formate to CO_2_^80^. CO_2_ can be assimilated through Calvin Cycle, the only C fixation pathway found in this genome and other known Methylomirabilales^80,85^. Accordingly, the Serine Cycle for formaldehyde assimilation is absent. Using enrichment cultures and carbon isotope tracing experiments, Rasigraf et al. (2014)^85^ demonstrated that the methanotrophic *M. oxyfera* assimilated exogenous 13 CO_2_, when provided with CH4, indicating autotrophic CO_2_ fixation. The question arises whether these non-methanotrophic methylotroph counterparts could also assimilate exogenous CO_2_ (and not only from the formaldehyde degradation). Considering the wide distribution of these organisms in the deep-sea sediments of the Santos Basin slope presented in our study, this could represent an overlooked primary production process in the area. However, experimental demonstration of this capability would be required.

Microorganisms inhabiting continental slope sediments may endure a wide range of conditions, including oligotrophic environments, low temperature, and high hydrostatic pressure (HHP). Such conditions may select organisms with suitable niches, and by exploring the metabolic potential of SB_MAG_00001 we could shed light into the ecology and adaptation of this microorganism. Living in oligotrophic sediments demands metabolic strategies to cope with scarce and oscillating resources. In this regard, methylotrophy might represent an advantageous trait, considering that C1 compounds are ubiquitous in the ocean^86–89^. In addition to methanol, other C1 compounds such as methane, methyl halides, methylated amines, and methylated sulphur compounds are also commonly found in the marine environment^89,90^. Studies have suggested the importance of methylotrophic microorganisms for the turnover these C1 compounds, and that microbial activity prevents their accumulation in marine sediments^91,92^.

In addition to carbon substrate, metabolic capabilities related to nutrient cycling can reveal ecological strategies to couple with the availability of reduced compounds and electron acceptors. For instance, SB_MAG_00001 contains genes for the first step of the dissimilatory nitrate reduction and denitrification (*nar*GH), pathways used as alternative respiratory pathways under low levels of oxygen by several microorganisms^93,94^. Furthermore, *nxr*AB, involved in nitrite oxidation, and the gene *hao*, which converts hydroxylamine to nitrite, are also present. As for sulphur cycling, genes related to assimilatory sulphate reduction (ASR) (*sat*, *cys*N, *cys*C, *sir*, *cys*K), and thiosulphate oxidation (*sox, doxD*) were annotated. ASR is a fundamental metabolic route, as sulphur is an essential element in all organisms present in biomolecules, such as amino acids^95^. In addition, thiosulphate can be an important intermediate in the sulphur cycle in marine sediments and it might be generated from the anoxic sulphide oxidation^96^.

Regarding phosphorus metabolism, genes encoding for polyphosphate kinase (PPK) and exopolyphosphatase (PPX) were detected. These enzymes are responsible for polyP accumulation and degradation, a trait that may confer an advantage in environments under high oscillation in phosphate availability and other stress types^97,98^. In addition, the presence of the gene for phosphonate transport (*phn*D) indicates a potential use of organic phosphorus. Besides *phn*D, other transporters provided some indications of the potential ecophysiology of this methylotroph, including transporters for Iron, glutamate/ aspartate, phospholipid/ cholesterol/ gamma-HCH, amino acids, and tungstate (Fig. 5).

Under deep-sea conditions, adaptations for psychrophilic and piezophilic lifestyles may confer advantages. Our sampling points ranged from 433 to 747 m deep where water temperatures can be as low as 5 °C for the Antarctic Intermediate Water mass^28^. Some traits, such as the expression of cold and shock proteins can have key roles in the cold seafloor^99^. SB_MAG_00001 contains *csp*A and *rhl*B genes, which encode for the cold-shock protein and cold-shock dead-box protein-A, respectively, and have been suggested to have a role in the adaptation to cold conditions^100,101^.

One of the major findings in cellular adaptations to high hydrostatic pressure conditions is the accumulation of solutes in the bacteria, which may play the role of a “piezolyte” acting as protein-stabilising solutes^102^. Accumulation of protein-stabilising solutes, such as β-hydroxybutyrate (PHB), is often observed in organisms living under HHP^94^. Polyhydroxyalkanoate synthase (*pha*C), a gene related to PHB biosynthesis, is present in SB_MAG_00001. Compatible solutes can confer resistance to multiple stresses, including hydrostatic and osmotic pressure^103^. Other genes related to compatible solutes were found in SB_MAG_00001: the *trk* system potassium uptake protein and the monovalent cation:H^+^ antiporter, and CPA1 family *mrp,* for sodium efflux. The genes for TRAP-type transport system periplasmic protein (*yiaO*) and trehalose synthesis (*ots*AB) seem to be present only in the CSP1-5 cluster, while the osmoprotectant transport system (*opuB*C) and the mechanosensitive channels (*ybi*O) are also found in the genomes of the methanotrophic Methylomirabilales.

## Concluding remarks

We studied the prokaryotic communities in sediments in pockmark and salt diapir fields from the Continental Slope in Santos Basin. Using a combination of metabarcoding and metagenomic approaches associated with phylogenetic tools, we observed that a non-methanotrophic methylotroph from the order Methylomirabilales was widespread across all samples and was the highest-quality MAG retrieved. We explored the metabolic potential of this genome (classified into the family CSP1–5) and described its methanol oxidising capability, along with several genes with the potential to improve the fitness of the organism in the deep-sea environment. The widespread nature of this organism suggests a potential important role of methanol metabolism in this continental slope area. We further argued whether it could contain an overlooked autotrophic CO_2_ fixation pathway.

The results also provide evidence that studies based only on metabarcoding may lead to misclassification of the members of the Methylomirabilales, which has profound relevance for the conclusions regarding the roles of the organisms in the environment, as the order contains methanotrophic (Methylomirabilaceae) and non-methanotrophic (CSP1–5) members. Ribosomal sequences misclassified as Methylomirabilaceae were retrieved from various environments, and, therefore, the relevance of methanol metabolism may be neglected in previous studies.

### Supplementary Information

Supplementary Information.

## Data Availability

16S rRNA gene sequencing data are available in the National Center for Biotechnology Information Sequence Read Archives under BioProject ID PRJNA818533. Metagenome raw sequences are available in the GenBank repository under BioProject ID PRJNA818670. The MAG SB_MAG_00001 was deposited in Figshare under https://doi.org/10.6084/m9.figshare.20080031.
