# Supplementary material for: Assessment of prokaryotic communities in Southwestern Atlantic deep-sea sediments reveals prevalent methanol-oxidising Methylomirabilales
Source: Sci Rep. 2023 Aug 7;13:12782. doi: 10.1038/s41598-023-39415-9 (PMC10406867; doi:10.1038/s41598-023-39415-9)
Supplement: Supplementary file 1 — Supplementary Information. [file 41598_2023_39415_MOESM1_ESM.pdf]

Supplemental Information for:

Assessment of prokaryotic communities in Southwestern Atlantic deep-sea sediments reveals prevalent methanol-oxidising Methylomirabilales

Francielli V. Peres, Fabiana S. Paula*, Amanda G. Bedia, Júlia B. Gontijo, Michel M. de Mahiques, Vivian H. Pellizari

*Corresponding author:
Fabiana S. Paula
E-mail address: fabianaspaula@gmail.com
Instituto Oceanográfico, Universidade de São Paulo, Praça do Oceanográfico, 191, São Paulo CEP: 05508-120, Brasil.

**Supplementary Tables**	**Page**
Supplementary Table 1	2
Supplementary Table 2	3
Supplementary Table 3	4
Supplementary Table 4	5
Supplementary Table 5	6
Supplementary Table 6	9
Supplementary Table 7	10
Supplementary Table 8	12

Tables S1. Sampling stations studied at Santos Basin continental slope and sample metadata.

Sample ID	Stratum (cm)[a]	Latitude	Longitude	Depth (m)[b]	Site[c]
Surface_255	0-2	26°29.677’S	45°58.153’W	652	Control
Subsurface_255	22-24	26°29.677’S	45°58.153’W	652	Control
Surface_258	0-2	26°15.715’S	45°40.97’W	543	Control
Subsurface _258	22-24	26°15.715’S	45°40.97’W	543	Control
Surface_250	0-2	26°49.590’S	46°24.27’W	433	Diapir
Subsurface _250	16-18	26°49.590’S	46°24.27’W	433	Diapir
Surface_259	0-2	26°33.776’S	46°07.008’W	559	Diapir
Subsurface _259	22-24	26°33.776’S	46°07.008’W	559	Diapir
Surface_260	0-2	26°33.710’S	46°07.470’W	517	Diapir
Subsurface _260	22-24	26°33.710’S	46°07.470’W	517	Diapir
Surface_253	0-2	26°14.72’S	45°40.97’W	730	Pockmark
Subsurface _253	22-24	26°14.72’S	45°40.97’W	730	Pockmark
Surface_254	0-2	26°15.715’S	45°42.971’W	747	Pockmark
Subsurface _254	22-24	26°15.715’S	45°42.971’W	747	Pockmark
Surface_257	0-2	26°32.075’S	46°04.822’W	698	Pockmark
Subsurface _257	22-24	26°32.075’S	46°04.822’W	698	Pockmark

[a] Sediment stratum (depth) selected from the core of sediment collected. Surface: 0-2 cm; Subsurface: 16-18 or 22-24 cm

[b] Water column depth

[c] Sampling site geomorphological feature

Tables S2. Richness and diversity of prokaryotes in surface and subsurface sediment samples.

**Sample ID**	**No. of Sequences**	**Observed ASV**	**Chao1**	**Shannon**
**Control Surface**				
Surface_255	25.853	744	776.354	6.136
Surface_258	49.839	1.094	1214.839	6.449
Sum	*75692*	*1.838*	*1991.193*	*12.585*
Mean ± SD	*37.846 ± 11.99*	*919 ± 175*	*995.59 ± 219.24*	*6.292 ± 0.156*
**Control Subsurface**				
Subsurface _255	39.456	1.176	1320.460	6.574
Subsurface _258	64.296	816	932.575	5.632
Sum	*103.752*	*1.992*	*3.277.522*	*12.206*
Mean ± SD	*51.976 ± 12.42*	*996 ± 180*	*1321.393 ± 659.76*	*6.103 ± 0.471*
**Diapir Surface**				
Surface_250	48.861	1.181	1288.013	6.548
Surface_259	38.970	921	971.281	6.247
Surface_260	36.265	999	1079.577	6.419
Sum	*124.096*	*3.101*	*3338.871*	*19.214*
Mean ± SD	*38.97 ± 5.41*	*999 ± 108.93*	*1079.577 ± 131.44*	*6.419 ± 0.123*
**Diapir Subsurface**				
Subsurface _250	42.091	1193	1348.472	6.537
Subsurface _259	32.897	917	992.607	6.224
Subsurface _260	38.272	721	790.538	5.785
Sum	*113.260*	*2831*	*3131617*	*18.546*
Mean ± SD	*38.272 ± 3.77*	*917 ± 193.64*	*992.607 ± 230.64*	*6.22 ± 0.308*
**Pockmark Surface**				
Surface_253	23.675	708	724.5541	6.158
Surface_254	6.972	292	293.500	5.292
Surface_257	38.816	932	1001.227	6.262
Sum	*69.463*	*1932*	*1294.727*	*11.554*
Mean ± SD	*23.67 ± 13.0*	*708 ± 265.16*	*1001.227 ± 3123.8*	*6.15 ± 0.434*
**Pockmark Subsurface**				
Subsurface _253	28.379	952	724.554	6.158
Subsurface _254	20.535	822	844.557	6.299
Subsurface _257	38.673	1.186	1295.572	6.556
Sum	*87.587*	*2.960*	*2864.683*	*19.013*
Mean ± SD	*28.379 ± 7.427*	*952 ± 150.61*	*844.557 ± 245.82*	*6.299 ± 0.164*

Table S3. Two-way ANOVA statistics evaluating the effects of strata and site on Chao1 and Shannon diversity indexes.

**Chao1**	**SS**	**df**	**MS**	**F**	**P**
(stratum) Surface:Subsurface	52896.80	1	52896.80	0.70	0.4206
(site) Control:Diapir:Pockmark	250570.95	2	125285.48	1.67	0.2326
stratum x site	84649.68	2	42324.84	0.56	0.5867
Error	825423.66	11	75038.51		
Total	1213541.09	16			
**Shannon**	**SS**	**df**	**MS**	**F**	**P**
(stratum) Surface:Subsurface	0.01	1	0.01	0.07	0.7962
(site) Control:Diapir:Pockmark	0.15	2	0.08	0.50	0.6197
stratum x site	0.50	2	0.25	1.67	0.2326
Error	1.65	11	0.15		
Total	2.31	1			

Site= Control, Diapir and Pockmark. Strata= Surface (0-2cm) and Subsurface (16-18cm or 22-24cm)

Table S4. Permutational analysis of variance (PERMANOVA) evaluating the effects of site and strata on community composition.

Factor	Df	SumsOfSqs	MeanSqs	F.Model	R^2	P value
Site	2	0.6665	0.33324	1.1750	0.11858	0.231
Strata	1	1.5204	1.52044	5.3611	0.27052	0.001
Site:Strata	2	0.5975	0.29875	1.0534	0.10631	0.360
Residuals	10	2.8361	0.28361	0.50459	0.35164	
Total	15	5.6205			1.00000	

Site= Control, Diapir and Pockmark. Strata= Surface (0-2cm) and Subsurface (16-18cm or 22-24cm)

Table S5. Core Microbiome analysis – list of ASVs shared by all samples (central), by surface samples only, and by subsurface samples only.

ASV_ID	Domain	Phyla	Class	Order	Family
**Central Core Microbiome**					
88ece184badcd461a1c452b9168238dd	Bacteria	Proteobacteria	Alphaproteobacteria	Rhizobiales	Hyphomicrobiaceae
d42457886a830872cbbecf93f40e7b7d	Bacteria	Methylomirabilota	Methylomirabilia	Methylomirabilales	Methylomirabilaceae
**Surface Core Microbiome**					
04013e82bfa1a8727a8d9230ad85f757	Bacteria	Proteobacteria	Alphaproteobacteria	Kiloniellales	Kiloniellaceae
0498ad663b7f79af564bbd917becf6a4	Archaea	Crenarchaeota	Nitrososphaeria	Nitrosopumilales	Nitrosopumilaceae
07f4ec67a67b6e689a8b1f3bc7043e83	Bacteria	Proteobacteria	Alphaproteobacteria	uncultured	uncultured
0b678d1f0f13f61e6383bb6e0948c798	Bacteria	Dadabacteria	Dadabacteriia	Dadabacteriales	Dadabacteriales
10b767aa94756f5887aeb2cb7fc9f2f4	Bacteria	Gemmatimonadota	PAUC43f	PAUC43f	PAUC43f
140a6b70a72fcedca42dad6d25f5fd98	Bacteria	Proteobacteria	Gammaproteobacteria	AT-s2-59	AT-s2-59
1c1edb73165695a5bd9ca622dc2b711f	Archaea	Crenarchaeota	Nitrososphaeria	Nitrosopumilales	Nitrosopumilaceae
1d1d67547136a33fcdfcd306f5702f6e	Bacteria	Proteobacteria	Alphaproteobacteria	Kiloniellales	Kiloniellaceae
20c3f85fb8a8652ec8b1330d433aed32	Bacteria	Proteobacteria	Alphaproteobacteria	Kiloniellales	Kiloniellaceae
21ace7371bd6993fc047f6187e925dbf	Bacteria	Proteobacteria	Alphaproteobacteria	Rhizobiales	Methyloligellaceae
25733a39b1dfa679b2d0712dff87f258	Bacteria	Proteobacteria	Gammaproteobacteria	MBMPE27	MBMPE27
2b78b7292871cab7dd6e4ab1518ef6a3	Bacteria	NB1-j	NB1-j	NB1-j	NB1-j
2d2d4c698a867a06755afc4dce823452	Bacteria	Gemmatimonadota	BD2-11	BD2-11	BD2-11
2d6449d9ef1747714ad9b8242947215b	Bacteria	Proteobacteria	Gammaproteobacteria	MBMPE27	MBMPE27
2e48c01cef7f4daf8c36285a101c0197	Bacteria	Proteobacteria	Alphaproteobacteria	-	-
2f898363289df017eb456cd4434e976e	Bacteria	Dadabacteria	Dadabacteriia	Dadabacteriales	Dadabacteriales
30d230e4e00057dfdf4adde04627529d	Bacteria	Proteobacteria	Gammaproteobacteria	Oceanospirillales	Nitrincolaceae
33885eacf84739fdf59109d9a30c4143	Bacteria	Proteobacteria	Alphaproteobacteria	-	-
33df8b69f75f0e4b605028dbfc54ea96	Archaea	Crenarchaeota	Nitrososphaeria	Nitrosopumilales	Nitrosopumilaceae
34bb86368caea06b45a8f23fea1dbc5c	Archaea	Crenarchaeota	Nitrososphaeria	Nitrosopumilales	Nitrosopumilaceae
3f1bc0782561cd249c71637d97a7738f	Archaea	Crenarchaeota	Nitrososphaeria	Nitrosopumilales	Nitrosopumilaceae
41a20373352d33616e961818dbb179e5	Bacteria	Proteobacteria	Gammaproteobacteria	Nitrosococcales	Nitrosococcaceae
4436051b8caa9b2f2ea3bdacd1afdeb9	Bacteria	Proteobacteria	Gammaproteobacteria	Steroidobacterales	Woeseiaceae
45ff6775638a7dc8be1a5966055555d2	Archaea	Crenarchaeota	Nitrososphaeria	Nitrosopumilales	Nitrosopumilaceae
471f8559df7c30dbc2b2d0a0aeed68ed	Bacteria	Nitrospirota	Nitrospiria	Nitrospirales	Nitrospiraceae
4c16b55ededa3ba039f6662cc72e63f6	Bacteria	Proteobacteria	Alphaproteobacteria	-	-

**Table S5** – continuation					
**ASV_ID**	**Domain**	**Phyla**	**Class**	**Order**	**Family**
502c02860f623e5ff8795422903cbfde	Bacteria	Acidobacteriota	Vicinamibacteria	Vicinamibacterales	uncultured
56bbc504d74551cd72635d16d0bac851	Bacteria	Proteobacteria	Alphaproteobacteria	Kiloniellales	Kiloniellaceae
57c59f2ccbca5b03a1e04670dd8f0010	Archaea	Crenarchaeota	Nitrososphaeria	Nitrosopumilales	Nitrosopumilaceae
5a304aed857826a705f0a14cefb2db9f	Bacteria	Proteobacteria	Gammaproteobacteria	AT-s2-59	AT-s2-59
63e33a7d7b5a21a9df845a4a05847bc9	Bacteria	Gemmatimonadota	BD2	BD2	BD2
65e42fc434451815d377d184a77fe1c7	Bacteria	Proteobacteria	Gammaproteobacteria	Steroidobacterales	Woeseiaceae
68c9a3dacdbfd3b63b79cb29e6673b4b	Bacteria	Proteobacteria	Gammaproteobacteria	Steroidobacterales	Woeseiaceae
6c8fe3f5b0b240007f0ffea2faeaae73	Bacteria	Proteobacteria	Alphaproteobacteria	Rhizobiales	KF-JG30-B3
6fd0f2ed0e237e81dcadcbab72284bde	Bacteria	Proteobacteria	Alphaproteobacteria	Kiloniellales	Kiloniellaceae
702e8b2107edfe1dbe47dba8bc5d185c	Bacteria	Proteobacteria	Gammaproteobacteria	Gammaproteobacteria	Unknown
7079b0bac05957134fe21be3636f405c	Bacteria	-	-	-	-
739d21ef1089dd8495682f60f7727ed9	Bacteria	Bacteroidota	Bacteroidia	Cytophagales	Cyclobacteriaceae
73a76d201ec82c666a150f6cfb7473c4	Bacteria	Proteobacteria	Gammaproteobacteria	Alteromonadales	Shewanellaceae
7485b488409ea7697e23e79766acdd86	Bacteria	Proteobacteria	Gammaproteobacteria	EPR3968-O8a-Bc78	EPR3968-O8a-Bc78
77701520d52299d7f15e2871502f1664	Bacteria	Planctomycetota	Pla3_lineage	Pla3_lineage	Pla3_lineage
7cac67fe6bbff3aeb2194c826f91f565	Bacteria	Proteobacteria	Alphaproteobacteria	-	-
7dcf93ffa46f9927ef6df7ab89a09613	Bacteria	Nitrospirota	Nitrospiria	Nitrospirales	Nitrospiraceae
7e7d25eb23ad32f12e195b377e4b37ba	Bacteria	Proteobacteria	Gammaproteobacteria	Burkholderiales	Nitrosomonadaceae
83365de7a4bd74406238426c3e5d34d2	Bacteria	Proteobacteria	Gammaproteobacteria	Nitrosococcales	Nitrosococcaceae
850e720604e35b0e152d821aa5391dc0	Bacteria	Proteobacteria	Alphaproteobacteria	Rhizobiales	Methyloligellaceae
8816d7893041e98f9cad4704883a48d8	Bacteria	Planctomycetota	Planctomycetes	Pirellulales	Pirellulaceae
8c504df61b282e1fa88491325c5e00d8	Bacteria	-	-	-	-
8cfb8c64de2d14262e2b7ba2da2b683b	Bacteria	Proteobacteria	Alphaproteobacteria	Kiloniellales	Kiloniellaceae
8f4c1632a796a6609c6368164542e423	Bacteria	Proteobacteria	Alphaproteobacteria	Kiloniellales	Kiloniellaceae
8f6ed589c9351e9b5f6b0ffe9e7c100e	Bacteria	Proteobacteria	Alphaproteobacteria	Defluviicoccales	uncultured
9843e7cd10cfbdd58b222aa2b44c6e37	Bacteria	Nitrospirota	Nitrospiria	Nitrospirales	Nitrospiraceae
9a1c885c36a0ece622a7f8f92c07cd7d	Bacteria	Acidobacteriota	AT-s3-28	AT-s3-28	AT-s3-28
9cb32e4ce15398ecb5d3c94f335eb296	Bacteria	NB1-j	NB1-j	NB1-j	NB1-j
9e5dcea274a19a7008d2265efff2923a	Bacteria	Proteobacteria	Gammaproteobacteria	EPR3968-O8a-Bc78	EPR3968-O8a-Bc78
a8c2c0f8a2812df378266e804e702aa9	Bacteria	Proteobacteria	Gammaproteobacteria	Gammaproteobacteria	Unknown

**Table S5** – continuation					
**ASV_ID**	**Domain**	**Phyla**	**Class**	**Order**	**Family**
aa834f0722681701e21903fa1b9329bb	Bacteria	Proteobacteria	Alphaproteobacteria	Kiloniellales	Kiloniellaceae
ab418e07af33521ba183bb4b836b5168	Archaea	Crenarchaeota	Nitrososphaeria	Nitrosopumilales	Nitrosopumilaceae
ab8701fe9bc69d2545f1d1841be43ca0	Bacteria	Proteobacteria	Gammaproteobacteria	Nitrosococcales	Nitrosococcaceae
add9c0f8a87fa265a195397a8d0d58b6	Archaea	Crenarchaeota	Nitrososphaeria	Nitrosopumilales	Nitrosopumilaceae
af562fbdd67ae04b1ae167190232b522	Bacteria	Proteobacteria	Alphaproteobacteria	-	-
b1cf9092b41e5db7ba632d91fd070bd8	Bacteria	Proteobacteria	Gammaproteobacteria	Gammaproteobacteria	Unknown
b713c9afb89b316e229a19441dff4d1b	Bacteria	Gemmatimonadota	PAUC43f	PAUC43f	PAUC43f
b7f91f8b2b617fadf4d321ca6eaf0313	Bacteria	NB1-j	NB1-j	NB1-j	NB1-j
b8748189b9c61925bb5f0eddb7b3e734	Bacteria	Gemmatimonadota	PAUC43f	PAUC43f	PAUC43f
bdc959f8622c41e5649e80f6989a239a	Archaea	Crenarchaeota	Nitrososphaeria	Nitrosopumilales	Nitrosopumilaceae
c1dd3fba8751fc4558951c6225bf78a9	Bacteria	Gemmatimonadota	BD2-11	BD2-11	BD2-11
c3e3dbe5247b1352b53856ff86aac1c1	Bacteria	Proteobacteria	Gammaproteobacteria	MBMPE27	MBMPE27
c3eefd9de45c9afc27757e0931c955b1	Archaea	Crenarchaeota	Nitrososphaeria	Nitrosopumilales	Nitrosopumilaceae
c67a44e7c182d82abd4d193f45a00d5c	Bacteria	Acidobacteriota	Subgroup_22	Subgroup_22	Subgroup_22
c8d97f24b030206f07d1c54b2a631393	Bacteria	NB1-j	NB1-j	NB1-j	NB1-j
d85f37f01b54e59aaa9b5194385dd433	Bacteria	Myxococcota	bacteriap25	bacteriap25	bacteriap25
dbefeb8a83c0e0cc2b9f8490f851e228	Bacteria	Proteobacteria	Gammaproteobacteria	MBMPE27	MBMPE27
e01197bd4113e43decca0c3663e67f43	Bacteria	Bacteroidota	Bacteroidia	Cytophagales	Cyclobacteriaceae
e5f6e798a12918409bf6fc6ae3bc509e	Archaea	Crenarchaeota	Nitrososphaeria	Nitrosopumilales	Nitrosopumilaceae
e6a468f5c87528890754b9440ce9c39b	Bacteria	Bacteroidota	Bacteroidia	Cytophagales	Cyclobacteriaceae
e7b305d64864e6b6342640ef7cd475c4	Bacteria	NB1-j	NB1-j	NB1-j	NB1-j
ed3e7227f2817d733a9c26b6a87af048	Bacteria	Proteobacteria	Alphaproteobacteria	Kiloniellales	Kiloniellaceae
ef63eb3f3f5a1749e2025c173dd78b1c	Bacteria	Planctomycetota	Planctomycetes	Pirellulales	Pirellulaceae
f33200e3502fdd17e126d1333fb55140	Bacteria	Bacteroidota	Bacteroidia	Flavobacteriales	Crocinitomicaceae
**Subsurface Core Microbiome**					
88ece184badcd461a1c452b9168238dd	Bacteria	Proteobacteria	Alphaproteobacteria	Rhizobiales	Hyphomicrobiaceae
d42457886a830872cbbecf93f40e7b7d	Bacteria	Methylomirabilota	Methylomirabilia	Methylomirabilales	Methylomirabilaceae

Table S6. List of MAGs recovered from the sediment samples, their taxonomic classification and quality parameters.

MAGs	Completeness (%)	Contamination (%)	Contig number	CG %	N50	Total length	GTDB-TK taxonomy
SB_MAG_00001	94.244	2.158	403	58.583	7968	2397160	Bacteria; Methylomirabilota; Methylomirabilis, CPS1-5; CPS1-5
SB_MAG_00002	90.647	2.877	323	57.953	9268	2379090	Bacteria; Nitrospirota; Nitrospiria; Nitrospirales; Nitrospiraceae
SB_MAG_00003	83.453	1.438	520	44.422	3070	1518180	Bacteria; Proteobacteria; Gammaproteobacteria; Burkholderiales; Nitrosomonadaceae; GCA2721545
SB_MAG_00004	82.014	2.158	928	57.198	3165	2823452	Bacteria; Proteobacteria; Gammaproteobacteria; Burkholderiales; Nitrosomonadaceae; GCA2721545
SB_MAG_00005	76.978	2.158	2.328	54.233	2573	5918495	Bacteria; Proteobacteria; Gammaproteobacteria; GCA-2729495; GCA-2729495; GCA2729495
SB_MAG_00006	72.661	0.719	1049	63.004	3646	3537226	Bacteria; Proteobacteria; Gammaproteobacteria; HK1
SB_MAG_00007	71.942	2.877	515	40.507	2826	3537226	Bacteria; Proteobacteria; Gammaproteobacteria; UBA4486; UBA4486
SB_MAG_00008	71.942	2.877	2024	53.166	2000	4170056	Bacteria; Binatota; Binatia; UBA9968
SB_MAG_00009	66.906	2.158	1984	62.711	2381	4744342	Bacteria; Proteobacteria; Gammaproteobacteria; HK1
SB_MAG_00010	63309	2.158	841	66363	1957	1698391	Bacteria; Proteobacteria; Alphaproteobacteria;Kiloniellales
SB_MAG_00011	60.431	2.158	2299	57.615	1864	4436461	Bacteria; Proteobacteria; Gammaproteobacteria;Woeseiales; Woeseiaceae;
SB_MAG_00012	58.992	1.438	963	66.436	2056	2034755	Bacteria; Proteobacteria; Alphaproteobacteria; Kiloniellales;Kiloniellaceae;
SB_MAG_00013	57.554	0.000	455	34.752	2205	1040817	Bacteria; Dadabacteria; UBA1144
SB_MAG_00014	53.956	1.438	3617	59.202	1889	7172570	Bacteria; Tectomicrobia; Entotheonellia; Entotheonellales
SB_MAG_00015	50.617	2.469	235	34.209	1951	475213	Archaea; Crenarchaeota;Nitrososphaeria;Nitrososphaerales; Nitrosopumilaceae;
SB_MAG_00016	51.234	3.703	336	33.915	2171	751328	Archaea; Crenarchaeota;Nitrososphaeria;Nitrososphaerales;Nitrosopumilaceae
SB_MAG_00017	50.000	2.469	172	33.744	3972	608712	Archaea;Crenarchaeota;Nitrososphaeria;Nitrososphaerales;Nitrosopumilaceae;CSP1-1
SB_MAG_00018	50.000	4.321	370	33.771	2163	819900	Archaea;Crenarchaeota;Nitrososphaeria;Nitrososphaerales;Nitrosopumilaceae
SB_MAG_00019	46.762	2.158	2042	66.128	1817	3842992	Bacteria;Myxococcota;UBA9160
SB_MAG_00020	46.043	2.877	638	66.476	1975	1299393	Bacteria;Proteobacteria;Alphaproteobacteria;Kiloniellales
SB_MAG_00021	43.884	1.438	691	48.608	1851	1322597	Bacteria; Nitrospirota;Nitrospiria;Nitrospirales;UBA8639;UBA8639
SB_MAG_00022	43.827	3.086	246	34.212	2190	557612	Archaea;Crenarchaeota;Nitrososphaeria;Nitrososphaerales;Nitrosopumilaceae
SB_MAG_00023	40.287	1.438	1466	65.685	1804	2742135	Bacteria;Proteobacteria;Alphaproteobacteria;UBA6615
SB_MAG_00024	40.123	1.851	128	34.001	3182	379099	Archaea;Crenarchaeota;Nitrososphaeria;Nitrososphaerales;Nitrosopumilaceae;CSP1-1
SB_MAG_00025	40.123	1.851	261	33.521	2319	615250	Archaea;Crenarchaeota;Nitrososphaeria;Nitrososphaerales;Nitrosopumilaceae;CSP1-1
SB_MAG_00026	38.848	0.719	608	56.672	2073	1308546	Bacteria;Proteobacteria;Gammaproteobacteria;Woeseiales;Woeseiaceae;SZUA-117
SB_MAG_00027	40.123	3.703	411	33.581	2092	892541	Archaea;Crenarchaeota;Nitrososphaeria;Nitrososphaerales;Nitrosopumilaceae
SB_MAG_00028	40.123	4.938	377	34.542	1974	767898	Archaea;Crenarchaeota;Nitrososphaeria;Nitrososphaerales;Nitrosopumilaceae
SB_MAG_00029	34.532	0.000	569	53.863	2121	1243523	Bacteria; Proteobacteria; Gammaproteobacteria; Acidiferrobacterales; SPGG2; SPGG2;
SB_MAG_00030	37.654	3.703	341	33.619	1973	704382	Archaea;Crenarchaeota;Nitrososphaeria;Nitrososphaerales;Nitrosopumilaceae
SB_MAG_00031	35.802	3.703	214	33.417	2826	579426	Archaea;Crenarchaeota;Nitrososphaeria;Nitrososphaerales;Nitrosopumilaceae;CSP1-1
SB_MAG_00032	30.246	1.234	184	33.889	2364	478985	Archaea;Crenarchaeota;Nitrososphaeria;Nitrososphaerales;Nitrosopumilaceae;CSP1-1
SB_MAG_00033	30.246	1.851	120	34.555	3043	346885	Archaea;Crenarchaeota;Nitrososphaeria;Nitrososphaerales;Nitrosopumilaceae
SB_MAG_00034	30.864	6.172	382	33.449	2081	814736	Archaea;Crenarchaeota;Nitrososphaeria;Nitrososphaerales;Nitrosopumilaceae

Table S7. Information on the sequences retrieved from Silva v138 database and used to build the phylogenetic tree.

Access number	Isolation source	Location information	Alignment Identity	DOI
AF317743	Cave-freshwater samples	Nullarbor region of Australia	100%	DOI: 10.1046/j.1462-2920.2001.00187.x
AM991193	Karst spring water - groundwater	Switzerland:Yverdon-les-Bains	87.82%	Unpublished
DQ906791	Subsurface soil	Oman	87.91%	Unpublished
EU335141	Saturated C horizon soil aggregate - Soil	Melton Branch Watershed, Oak Ridge, TN.	94.83%	DOI: 10.1128/AEM.01787-07
EU335144	Saturated C horizon soil aggregate	Melton Branch Watershed, Oak Ridge, TN.	87.66%	DOI: 10.1128/AEM.01787-07
EU491346	seafloor lavas from Hawai'i South Point X3	East Pacific Rise (EPR)	95.14%	DOI: 10.1038/nature06899
EU491462	Seafloor lavas from Hawai'i South Point X3	East Pacific Rise (EPR)	94.04%	DOI: 10.1038/nature06899
FJ205359	Deep marine sediments, depth:2725m	Pacific Ocean: Lau Basin	94.04%	Unpublished
GU363024	marine sediment from the South China Sea	South China Sea	97.11%	Unpublished
HM186756	Saturated zone of the Hanford Site 300 Area subsurface - River sediment	Hanford Site 300 Area near Richland, Washington state (USA)	93.79%	DOI: 10.1111 / j.1462-2920.2011.02659.x
HM186887	Saturated zone of the Hanford Site 300 Area subsurface - River sediment	Washington - EUA	97.13%	DOI: 10.1111 / j.1462-2920.2011.02659.x
HM186966	Saturated zone of the Hanford Site 300 Area subsurface - River sediment	Washington - EUA	94,29%	DOI: 10.1111 / j.1462-2920.2011.02659.x
HM187212	Saturated zone of the Hanford Site 300 Area subsurface - River sediment	Washington - EUA	87,88%	DOI: 10.1111 / j.1462-2920.2011.02659.x
HM187376	Saturated zone of the Hanford Site 300 Area subsurface - River sediment	Washington - EUA	91,11%	DOI: 10.1111 / j.1462-2920.2011.02659.x
JF265987	White microbial mat from lava tube wall - Cave	Portugal: Gruta da Malha, Terceira, Azores	94.90 %	DOI: 10.1089 / ast.2010.0562
JF747701	Underwater Cave - hypersaline groundwater	Dominican Republic - Manantial del Toro	95.14 %	Unpublished
JF747703	Underwater Cave - hypersaline groundwater	Dominican Republic - Manantial del Toro	94.82 %	Unpublished
JF809696	Hypersaline basin - Deep-Sea Hypersaline Sediments	Medea - Mediterranean Sea	94.23 %	DOI: 10.1264 / jsme2.ME12045
JN229988	Subseafloor sediment deeply buried coral carbonates and sediments	Porcupine Seabight – Atlantic Ocean	95.52 %	Unpublished
JN615730	Tan microbial mat from lava tube wall - Cave	Gruta da Malha, Terceira, Azores - Portugal	93.97 %	Unpublished
JN701089	Yellow microbial mat from lava tube wall- Cave	Gruta Madre de Deus Terceira, Azores - Portugal	97.83 %	Unpublished
JN886867	Carbonate sediments	Southwest Indian Ridge	94.16 %	Unpublished
JQ425959	saline Soil layer (0-10 cm)	Old Texcoco lake - Mexico	93.88 %	Unpublished
JX222001	Subsurface aquifer sediment	USA:Rifle - Colorado	93.31 %	DOI: 10.1111/j.1574-6941.2012.01363.x
JX227602	Sediment collected from station WS0902 deep-sea polymetallic nodules and the surrounding sediments	Clarion-Clipperton Fracture Zone – Pacific Ocean	95.34%	DOI: 10.1016 / j.dsr.2013.05.004

**Table S7.** continuation				
**Access number**	**Isolation source**	**Location information**	**Alignment Identity**	**DOI**
KM071663	Deep-sea hydrothermal vent sediments	East Pacific Ocean	93.38 %	Unpublished
KM454246	Marine sediment	Maluku Strait - Indonesian	96.83 %	Unpublished
KT223295	Slope sediment - a depth of 1250 m	Orca Basin - Gulf of Mexico	89.91 %	DOI: 10.30564 / jasr.v2i2.930
KT223298	Slope sediment	Orca Basin - Gulf of Mexico	91.25%	Unpublished
KT748567	Seafloor basalt (3 km depth)	Dorado Outcrop, East Pacific Rise	93.19 %	DOI: 10.3389/fmicb.2015.01470
KX172753	marine Sediment	Gulf of Mexico	97.71 %	DOI: 10.3389/fmicb.2016.01384
KY609420	Fe-Si-rich low-temperature hydrothermal precipitates	Lau Basin - Australia-Pacific	91.07 %	DOI: 10.1111 / j.1574-6941.2012.01367.x
LDXP01000044	Sediment at 5m depth<br>aquifer adjacent to the Colorado River	Rifle, Colorado - USA	84.87 %	DOI: 10.1111 / 1462-2920.12930
LXTG01000049	Sediment sample at the water-sediment interface<br>Oxic Deep-Sea Sediments	Central gyre - Pacific Ocean	92.81 %	DOI: 10.1128/AEM.01023-16.

Table S8. List of genes used in the metabolic reconstruction model (Figure 5). Predicted protein, Kegg ID and method where the gene was detected are shown.

Gene	Predicted protein	Kegg ID	Detected by		
			DRAM	Pangenome	HMMER Search
xoxF<br>mxaJ	lanthanide-dependent methanol dehydrogenase<br>mxaJ protein	K23995<br>K16254	x<br>x	x	
fae	5,6,7,8-tetrahydromethanopterin hydrolyase	K10713		x	x
fdoH	formate dehydrogenase iron-sulfur subunit	K00124		x	
mtdB	methylene-tetrahydromethanopterin dehydrogenase	K10714	x	x	
mch	methenyl-tetrahydromethanopterin cyclohydrolase	K01499	x	x	x
ftr	formylmethanofuran-tetrahydromethanopterin N-formyltransferase	K00672	x	x	x
fwdA<br>fmdA	formylmethanofuran dehydrogenase	K00200		x	
npd	nitronate monooxygenase	K00459	x	x	
hao	hydroxylamine dehydrogenase	K10535	x	x	
narGH	nitrate reductase	K00370<br>K00371	x	x	x
nxrAB	nitrite oxidoreductase	K00370<br>K00371			x
glnA	glutamine synthetase	K01915	x	x	
gltB	glutamate synthase (NADPH) large chain	K00265		x	
doxD	thiosulfate dehydrogenase (quinone) large subunit	K16937	x		
soxC	sulfane dehydrogenase	K17225	x		x
sat	sulfate adenylyltransferase	K00958		x	x
cysN	sulfate adenylyltransferase	K00956			x

Table S8. continuation					
**Gene**	**Predicted protein**	**Kegg ID**	**Detected by**		
cysC	adenylyl-sulfate kinase / sulfate adenylyltransferase	K00860		x	x
sir	sulfite reductase (ferredoxin)	K00392		x	
cysK	cysteine synthase	K01738	x	x	
			**DRAM**	**Pangenome**	**HMMER Search**
trK	potassium uptake protein	K03498<br>K03499	x	x	
otsA	trehalose 6-phosphate synthase	K00697	x		
otsB	trehalose 6-phosphate phosphatase	K01087	x	x	
ybiO	moderate conductance mechanosensitive channel	K22044	x	x	
phaC	poly[(R)-3-hydroxyalkanoate] polymerase	K03821	x	x	
yiaO	TRAP-type transport system periplasmic protein	K21395	x	x	
opuABC	osmoprotectant transport system	K05847<br>K05846<br>K05845	x	x	
cspA	cold shock protein	K03704	x	x	
rhlB	ATP-dependent RNA helicase	K03732	x	x	
ppx	exopolyphosphatase	K01524	x	x	
ppk	polyphosphate kinase	K00937	x	x	
phnD	phosphonate transport system substrate-binding protein	K02044	x		
afuABC	iron(III) transport system	K02012<br>K02011<br>K02010	x	x	
tupAC	tungstate transport system	K05772<br>K06857	x	x	

**Table S8.** continuation					
**Gene**	**Predicted protein**	**Kegg ID**	**Detected by**		
lolDCE	lipoprotein-releasing system	K09810<br>K09808	x	x	
lptBFG	lipopolysaccharide export system	K06861<br>K07091<br>K11720	x	x	
			**DRAM**	**Pangenome**	**HMMER Search**
mlaCDEF	phospholipid transport system	K07323<br>K02067<br>K02066<br>K02065	x	x	
nodJ	lipooligosaccharide transport system permease protein	K09694	x	x	
livFGHKM	branched-chain amino acid transport system	K01996<br>K01995<br>K01997<br>K01999<br>K01998	x	x	
